# AD|ARC (Administrative Data Agri-Research Collection): Comparative analysis of highest educational qualification in Welsh households receiving a farming subsidy with rurality-matched control households, using 2011 UK Census data.

**DOI:** 10.23889/ijpds.v9i5.2541

**Published:** 2024-09-18

**Authors:** Katy Addison, Sian Morrison-Rees, Paul Caskie, Liam Crowley, Laura Madden, Sean Scully

**Affiliations:** 1 Welsh Government; 2 Swansea University; 3 Sustainable Agri-Food Science Directorate, Agri-Food and Bioscience Institute (AFBI)

## Objective

To investigate highest level of educational attainment in farming households compared to rural non-farming households in Wales.

## Approach

We used the AD|ARC dataset of households in Wales receiving farming subsidies in 2010.

We included farming households containing at least one member reporting an Agricultural Main Occupation (AMO) and households where all members reported Non-Agricultural Main Occupations (Non-AMO) but farming occurs.

Utilising Census 2011 variables relating to age, gender, education and occupation we conducted comparative analyses of educational attainment within AMO, Non-AMO and non-farming rural households at both an individual and household level.

## Results

Findings will be presented comparing the educational attainment for AMO individuals with matched individuals within Non-AMO and non-farming rural households. At the household level, we calculated the highest level of education attained within each household and will present results comparing the highest household level of education between each group. We will present detailed results regarding the structure and educational attainment within these households.

Full results and findings will be presented at the conference but are currently subject to information governance restrictions.

## Conclusions

One indicator of resilience amongst farmers and farm households is ability to respond to economic and non-economic pressures by adopting new practices and techniques or reducing reliance on farming via diversification or off-farm employment. As educational attainment is closely correlated to acquisition of skills and is a gateway to employment, understanding relative and absolute levels of attainment is important in identifying need and devising policies which support agricultural and rural communities.

